# Traditional and non-traditional risk factors for peripheral artery disease development/progression in patients with type 2 diabetes: the Rio de Janeiro type 2 diabetes cohort study

**DOI:** 10.1186/s12933-021-01249-y

**Published:** 2021-02-27

**Authors:** Claudia R. L. Cardoso, Juliana V. Melo, Thainá R. M. Santos, Nathalie C. Leite, Gil F. Salles

**Affiliations:** 1 Department of Internal Medicine, University Hospital Clementino Fraga Filho, School of Medicine, Universidade Federal do Rio de Janeiro, Rua Croton, 72, Jacarepagua, Rio de Janeiro, RJ CEP: 22750-240 Brazil; 2 Department of Occupational Therapy, University Hospital Clementino Fraga Filho, School of Medicine, Universidade Federal do Rio de Janeiro, Rio de Janeiro, Brazil

**Keywords:** Aortic stiffness, Carotid intima-media thickness, Peripheral artery disease, Risk factors, Type 2 diabetes

## Abstract

**Background:**

The prognostic importance of non-traditional risk factors for peripheral artery disease (PAD) development/progression is scarcely studied in diabetes. We investigated if carotid intima-media thickness (CIMT) and carotid-femoral pulse wave velocity (cf-PWV) added prognostic information beyond traditional cardiovascular risk markers for PAD outcomes.

**Methods:**

Ankle-brachial index (ABI) was measured at baseline and after a median of 91 months of follow-up in 681 individuals with type 2 diabetes. Multivariate Cox regressions examined the associations between the candidate variables and the outcome. PAD development/progression was defined by a reduction in ABI ≥ 0.15 (to a level < 0.9) or limb revascularization procedures, lower-extremity amputations or death due to PAD. The improvement in risk discrimination was assessed by increases in C-statistics of the models.

**Results:**

Seventy-seven patients developed/progressed PAD: 50 reduced ABI to < 0.9, seven had lower-limb revascularizations, and 20 had amputations or death. Age, male sex, diabetes duration, presence of microvascular complications (peripheral neuropathy and diabetic kidney disease), baseline HbA_1c_, 24-h systolic BP (SBP) and mean cumulative office SBP and LDL-cholesterol were associated with PAD development/progression in several models. CIMT and cf-PWV were additionally associated with PAD outcomes, and their inclusion further improved risk discrimination (with C-statistic increases between 0.025 and 0.030). The inclusion of ambulatory 24-h SBP, instead of office SBP, also improved PAD risk discrimination.

**Conclusions:**

Increased CIMT and aortic stiffness are associated with greater risks of developing/progressing PAD, beyond traditional risk factors, in type 2 diabetes.

## Introduction

Diabetes is a strong risk factor for peripheral artery disease (PAD) development, with relative risks varying from two- up to fourfold in contrast to individuals without diabetes [1]. Subjects with diabetes and PAD have increased risks of adverse cardiovascular outcomes and mortality [2–4], as well as, PAD is associated with a great burden of morbidity and reduction in life quality [5].

Besides diabetes, several other traditional cardiovascular risk factors have been associated with PAD development and progression, including older age, tobacco smoking, arterial hypertension and dyslipidemia [6]. Some studies have investigated risk factors for PAD development specifically in diabetes [7–14]. Diabetes duration [7, 9, 12] and glycemic control have been shown as risk factors in some of them [7, 12, 13]. Additionally, the presence of microvascular complications, more specifically retinal photocoagulation therapy and macroalbuminuria, was associated with greater chances of PAD development in individuals from the ADVANCE study [14].

However, the prognostic importance of non-traditional risk factors for PAD development is less well investigated, particularly in diabetes [9]. Carotid intima-media thickness (CIMT) is regarded as a marker of subclinical atherosclerosis. A population-based study demonstrated that CIMT was a predictor of PAD incidence in type 2 diabetes [9]. Moreover, no previous study investigated if increased aortic stiffness, another established marker of subclinical cardiovascular disease [15], and also an independent risk factor for major adverse cardiovascular complications in type 2 diabetes [16, 17], could predict PAD development/progression.

Therefore, we aimed to investigate the factors associated with development or progression of PAD in a prospective long-term follow-up cohort of type 2 diabetic patients. We paid special attention to determine if CIMT and aortic stiffness were able to add prognostic information beyond traditional cardiovascular risk markers; and whether the mean cumulative exposure during follow-up of some factors, such as blood pressures (BPs), serum lipids and glycated hemoglobin (HbA_1c_), provided better prognostic information than their respective baseline values.

## Patients and methods

### Patients and baseline procedures

This was a prospective study, the Rio-T2D Cohort Study, with 750 patients with type 2 diabetes enrolled between August 2004 and December 2008 and followed-up until December 2019 in the diabetes outpatient clinic of our tertiary-care University Hospital. All participants gave written informed consent, and the local Ethics Committee had previously approved the study protocol. The characteristics of this cohort, the baseline procedures and the diagnostic definitions have been detailed elsewhere [4, 16, 18–20]. In brief, inclusion criteria were all adult type 2 diabetic individual up to 80 years old with either any microvascular (retinopathy, nephropathy or neuropathy) or macrovascular (coronary, cerebrovascular or peripheral artery disease) complication, or with at least two other modifiable cardiovascular risk factors (hypertension, dyslipidemia or smoking). Exclusion criteria were morbid obesity (body mass index ≥ 40 kg/m^2^), advanced renal failure (serum creatinine > 180 μmol/L or estimated glomerular filtration rate < 30 mL/min/1.73m^2^) or the presence of any serious concomitant disease limiting life expectancy. All were submitted to a standard baseline protocol that included a thorough clinical examination, including ankle-brachial index (ABI) measurement, a laboratory evaluation, and a 24-h ambulatory BP monitoring (ABPM). Diagnostic criteria for diabetic chronic complications were detailed previously [4, 16, 18–20]. In summary, coronary heart disease was diagnosed by clinical, electrocardiographic criteria, or by positive ischemic stress tests; and cerebrovascular disease by history and physical examination. The diagnosis of nephropathy needed at least two albuminurias ≥ 30 mg/24 h or confirmed reduction of glomerular filtration rate (eGFR ≤ 60 mL/min/1.73m^2^, estimated by the CKD-EPI equation, or serum creatinine > 130 μmol/L). Peripheral neuropathy was determined by clinical examination (knee and ankle reflex activities, feet sensation with the Semmes–Weinstein monofilament, vibration with a 128-Hz tuning fork, pinprick and temperature sensations) and neuropathic symptoms were assessed by a standard validated questionnaire [19]. Diabetic retinopathy, either background non-proliferative or proliferative, was determined by a complete ophthalmologic examination performed by a single retinal specialist [20]. Clinic blood pressure (BP) was measured three times using a digital oscillometric BP monitor (HEM-907XL, Omron Healthcare, Kyoto, Japan) with a suitable sized cuff on two occasions two weeks apart at study entry. The first measure of each visit was discarded and BP considered was the mean between the last two readings of each visit. Arterial hypertension was diagnosed if mean systolic (SBP) ≥ 140 mmHg or diastolic BP (DBP) ≥ 90 mmHg or if anti-hypertensive drugs had been prescribed. ABPM was recorded in the following month using Mobil-O-Graph, version 12 equipment (Dynamapa, Cardios LTDA., São Paulo, Brazil), and average 24-h SBP and DBP were registered (20). Laboratory evaluation included fasting glycemia, HbA_1c_, serum creatinine and lipids. Albuminuria was evaluated in two non-consecutive sterile 24-h urine collections. For this specific study, 68 individuals with clinical PAD, defined by a history of typical intermittent claudication, or previous limb revascularization procedures, foot ulceration or lower-extremity amputations, and one participant with a baseline ABI ≥ 1.30, were excluded, totaling 681 individuals analyzed. The patients were followed-up regularly at least 3–4 times a year until December 2019. Laboratory examinations were repeated 2–4 times a year during follow-up, except albuminuria that was repeated once annually. Mean cumulative values of BPs and of laboratory exams were recorded during the time interval between the 2 ABI measurements or any PAD outcome occurrence.

### ABI measurements and assessment of development or progression of PAD

After resting supine for at least 5 min, two BP readings were taken sequentially from each brachial and posterior tibial arteries (total of 8 measurements, 4 brachial and 4 tibial), using the same digital oscillometric BP monitor (HEM-907XL, Omron Healthcare, Kyoto, Japan), validated for ABI measurement [4, 21]. The BP monitors were pre-programmed to automatically record 2 BP readings with a 3 min interval from each limb. ABI was calculated as the lowest tibial BP in either leg divided by the highest brachial BP in either arm [22, 23]. Two ABI measurements were performed at baseline and after a median of 91 months (interquartile range 56–121 months) by a single independent examiner who was unaware of other clinical data. PAD incidence/progression was defined by a reduction in ABI of ≥ 0.15 to a level of at least < 0.9 [12, 13] or by the occurrence of any hard PAD outcome (limb revascularization procedures, amputations above the ankle or death due to PAD), whichever came first.

### Aortic stiffness measurement

Immediately after the 24-h ABPM recording, a single trained independent observer unaware of other patients’ data measured pulse wave velocity (PWV) along the descending thoraco-abdominal aorta (aortic stiffness) [15, 16], using the validated [24] foot-to-foot velocity method with the Complior equipment (Artech-Medical, France). Briefly, waveforms were obtained transcutaneously over the right common carotid and femoral arteries simultaneously during a minimum period of 10 to 15 s. The time delay (t) was measured between the feet of the 2 waveforms and the distance (D) covered by the waves was measured directly between femoral and carotid recording sites. Carotid-femoral PWV (cf-PWV) was calculated as D (m)/t (s). Three consecutive readings were obtained and cf-PWV considered was the mean between them. We used the recommended scaling factor of 0.8 to convert cf-PWV obtained using direct distances to ‘real’ cf-PWV [15]. The cut-off value for considering increased aortic PWV was > 10 m/s [15]. No participant had clinical aorto-iliac occlusive disease, which could falsely decrease cf-PWV. We had previously reported the reproducibility of cf-PWV measurements in our laboratory [25].

### Carotid ultrasound imaging

A detailed description of carotid ultrasound measuring methods is available elsewhere [18]. In brief, a single experienced vascular radiologist, unaware of other participants’ data, performed all carotid ultrasound studies with a high resolution B-mode ultrasound (Sonoline G40, Siemens, Munich, Germany) and a7.5 MHz linear array transducer. Carotid scanning measurements protocol was that recommended by the Manheim carotid intima-media thickness (CIMT) consensus [26] and by the American Society of Echocardiography [27]. Far-wall CIMT was measured bilaterally at the common carotid artery (20 to 60 mm from the flow divider), and mean values were registered. Increased CIMT was defined as > 1.05 mm (the median value). The reproducibility of CIMT measurements in our laboratory has been previously reported [28].

### Statistical analyses

Continuous data were described as means (SDs) or as medians (interquartile ranges [IQR]). Baseline characteristics of patients according to development/progression of peripheral artery disease were compared by t-test, Mann–Whitney or χ^2^ tests, when appropriate. The independent predictors of the PAD endpoint occurrence were examined by multivariate time-to-event Cox regressions (the time-interval between ABI examinations were considered as the time-to-event of participants with and without ABI reductions and the actual time-to-event for those with hard PAD outcomes). Candidate variables to enter the multivariate Cox analysis in Model 1 were the following: age, sex, body mass index (BMI), diabetes duration, smoking status, physical activity, diabetic treatment (metformin and insulin), anti-hypertensive treatment (number and classes of drugs), baseline office systolic and diastolic BPs, presence of macrovascular and microvascular complications at baseline, baseline HbA_1c_, serum lipids (HDL- and LDL-cholesterol and triglycerides), use of statins and aspirin, and C-reactive protein. A forward selection procedure was used to select the independent predictors, with a p-value < 0.10 as the criterion to enter and to remain into the model. Particularly for the presence of microvascular complications, further baseline models were fitted with each specific complication (diabetic kidney disease was further separated into microalbuminuria and decreased eGFR). A second model (Model 2) was fitted with the same candidate covariates, except that ambulatory BPs substituted office BPs. Finally, a third model (Model 3) included mean cumulative values of office BPs, HbA_1c_ and serum lipids measured until the 2nd ABI measurement or occurrence of a hard PAD outcome, instead of their baseline values. Regardless of their significance, age, sex, and the baseline ABI were forced into all models. The improvement in risk discrimination among these models was assessed by the C-statistic (analogous to the area under ROC curve applied to a time-to-event analysis), compared by the DeLong method [29]. The independent associations between cf-PWV and CIMT (analyzed both as continuous and as categorical variables, separated and concomitantly included) with PAD outcomes were evaluated by including them in previous Models 1, 2 and 3, with their improvements in risk discrimination assessed by the same C-statistic method. Two additional sensitivity analyses were performed: one excluding those clinically-asymptomatic individuals with baseline ABI < 0.9 and another excluding those individuals with any PAD outcome in the first 2-years of follow-up (to address possible reverse causality). All the Cox regression results were presented as hazard ratios (HRs) with their 95% confidence intervals (CIs) and a 2-tailed p-value < 0.05 was considered significant. Statistics were performed with SPSS version 19.0 (SPSS Inc, Chicago, Il., USA).

## Results

### Characteristics according to incidence/progression of peripheral arterial disease during follow-up

The mean time-interval between the 2 ABI measurements or the occurrence of a hard PAD outcome was 91 months (SD: 40 months, range from 4 to 189 months). Mean baseline ABI was 1.07 (SD: 0.09) and 2nd measurement was 1.03 (SD: 0.14). Overall, 77 patients had new incident or progressed PAD: 50 reduced ABI to < 0.9 (with reductions ≥ 0.15), seven were submitted to lower-limb revascularization procedures, and 20 had amputations above the ankle or death from PAD. Table 1 outlines the clinical and laboratory characteristics of all patients and of those with and without incident/progressive PAD during follow-up. Patients who developed/progressed PAD were older, more frequently past or current smokers, had greater diabetes duration and had higher prevalences of microvascular complications than those who did not developed/progressed PAD. They also had higher SBPs, although they used more anti-hypertensive medications, and higher LDL-cholesterol and albuminuria and lower eGFR than those who did not had PAD outcomes. Finally, patients who developed/progressed PAD had higher aortic stiffness and CIMT than those who did not.

**Table 1:** Baseline characteristics of all diabetic patients and divided according to the absence or presence of incidence/progression of peripheral artery disease (incidence defined as new ABI ≤ 0.90, and progression as a decrease in ABI > 0.15 in those with a basal ABI ≤ 0.90, or any lower limb revascularization procedure, any amputation above the ankle and death from PAD)

Characteristics	All patients (n = 681)	Patients without incidence/progression of PAD (n = 604)	Patients with incidence/progression of PAD (n = 77)	p-value
Age (years)	59.9 (9.6)	59.6 (9.7)	62.6 (8.4)	0.005
Male sex (%)	39.7	38.7	43.4	0.25
Body mass index (kg/m^2^)	29.7 (4.8)	29.8 (4.9)	29.1 (4.7)	0.23
Smoking, current/past (%)	44.8	43.5	55.3	0.066
Physical activity (% active)	22.3	22.6	19.7	0.56
Diabetes duration (years)	8 (3–15)	8 (3–15)	11 (6–20)	< 0.001
Chronic diabetic complications (%)				
Cerebrovascular disease	9.0	8.9	9.2	0.93
Coronary artery disease	15.3	14.7	19.7	0.30
Retinopathy	32.6	30.7	47.4	0.004
Nephropathy	31.5	29.1	50.0	< 0.001
Peripheral neuropathy	29	26.5	50.0	< 0.001
Diabetes treatment (%)				
Metformin	88.1	89.1	80.3	0.037
Sulfonylureas	42.9	42.6	44.7	0.73
Insulin	48.5	47.6	55.3	0.23
Aspirin	90.0	88.9	98.7	0.007
Dyslipidemia (%)	87.4	87.1	89.5	0.55
Statins use (%)	77.2	77.4	76.3	0.88
Arterial hypertension (%)	86.5	86.0	90.8	0.25
Anti-hypertensive treatment				
Number of drugs (%)	3 (1–3)	3 (1–3)	3 (2–4)	0.22
ACE inhibitors/AR blockers (%)	81.4	80.2	90.8	0.025
Diuretics (%)	62.7	61.5	72.4	0.064
Beta-blockers (%)	45.8	45.6	47.4	0.77
Calcium channel blockers (%)	28.2	27.4	34.2	0.22
Blood pressures (mmHg)				
Office SBP	147 (25)	146 (24)	154 (25)	0.009
Office DBP	84 (13)	84 (13)	83 (12)	0.46
Ambulatory 24 h SBP	128 (15)	128 (15)	134 (15)	0.001
Ambulatory 24 h DBP	74 (10)	74 (10)	74 (9)	0.50
Cumulative mean office SBP	140 (16)	140 (16)	146 (16)	0.001
Cumulative mean office DBP	78 (10)	78 (10)	76 (9)	0.14
Laboratory variables				
Fasting glycemia (mmol/L)	8.9 (3.8)	8.9 (3.8)	9.1 (4.0)	0.72
Baseline HbA_1c_ (%)	8.0 (1.9)	8.0 (1.9)	8.2 (2.2)	0.45
(mmol/mol)	63.9 (12.9)	63.9 (12.9)	66.1 (16.2)	
Cumulative mean HbA_1c_ (%)	7.8 (1.4)	7.8 (1.4)	7.9 (1.4)	0.52
(mmol/mol)	61.7 (11.1)	61.7 (11.1)	62.8 (11.1)	
Triacylglycerol (mmol/L)	2.0 (1.5)	2.0 (1.5)	2.1 (1.4)	0.65
Baseline HDL-cholesterol (mmol/L)	1.1 (0.3)	1.1 (0.3)	1.1 (0.3)	0.25
Cumulative mean HDL-cholesterol (mmol/L)	1.2 (0.3)	1.2 (0.3)	1.1 (0.3)	0.43
Baseline LDL-cholesterol (mmol/L)	3.0 (1.0)	3.0 (1.0)	3.3 (0.9)	0.008
Cumulative mean LDL-cholesterol (mmol/L)	2.5 (0.7)	2.6 (0.7)	2.7 (0.6)	0.16
C-reactive protein (mg/L)	2.8 (1.2–6.3)	2.9 (1.2–6.4)	2.4 (1.3–4.9)	0.34
Glomerular filtration rate (mL/min/1.73 m^2^)	81 (20)	82 (20)	75 (21)	0.005
Albuminuria (mg/24 h)	13 (7–41)	13 (7–38)	21 (9–74)	0.010
Time interval between ABI evaluations (months)	91 (40)	90 (41)	100 (36)	0.027
Baseline ABI	1.07 (0.09)	1.07 (0.09)	1.04 (0.11)	0.058
Follow-up ABI	1.03 (0.14)	1.07 (0.09)	0.77 (0.20)	< 0.001
Aortic stiffness (cf-PWV, ms/s)	10.9 (2.4)	10.8 (2.2)	12.5 (2.9)	< 0.001
Increased aortic stiffness (> 10 m/s, %)	29.0	26.3	48.6	< 0.001
CC-IMT (mm)	1.05 (0.16)	1.04 (0.15)	1.13 (0.18)	< 0.001

Values are proportions, and means (standard deviations) or medians (interquartile range)

*SBP* systolic blood pressure, *DBP* diastolic blood pressure, *HbA*_*1c*_ glycated hemoglobin, *HDL* high-density lipoprotein, *LDL* low-density lipoprotein, *ABI* ankle-brachial index, *cf-PWV* carotid-femoral pulse wave velocity, *CC-IMT* common carotid intima-media thickness

### Independent risk factors of peripheral artery disease incidence/progression

Table 2 shows the results of the multivariate Cox analysis for the covariates independently associated with PAD incidence/progression. On the first model, including baseline variables and office BPs, older age, longer diabetes duration, presence of microvascular complications and higher HbA_1c_ were independently associated with the outcome, whereas office SBP was borderline associated. In the model that included ambulatory BPs instead of office BPs (Model 2), age, male sex, diabetes duration, presence of microvascular complications, HbA_1c_ and 24-h SBP were independent predictors of PAD development/progression. In Model 3, using mean cumulative values of office BPs, serum lipids and HbA_1c_ until the occurrence of a hard PAD outcome or the 2nd ABI measurement, male sex, diabetes duration, presence of macro- and microvascular complications, and mean cumulative SBP and LDL-cholesterol were the independent predictors of PAD outcomes. Only Model 2 improved risk discrimination over baseline Model 1. Regarding the importance of distinct microvascular complications, the presence of diabetic kidney disease was an independent predictor of PAD outcomes in all models with HRs between 2.7 and 3.1 (both increased albuminuria and decreased eGFR were equivalent predictors). Peripheral neuropathy was also predictive of PAD in all models (HRs: 1.9–2.1); and retinopathy, either non-proliferative or proliferative or both, was not an independent predictor in any of the models (HRs between 1.4 and 1.5, p-values 0.13–0.20). Table 3 presents the results of multivariate Cox regressions, adjusted for the same variables as in Table 2, for the independent associations between aortic stiffness and CIMT with PAD development/progression. Carotid-femoral PWV, analyzed as a continuous variable, was independently associated with PAD outcomes in all models, but its inclusion improved risk discrimination only when simultaneously included with CIMT. Analyzed as a dichotomical variable (cf-PWV > 10 m/s), it only predicted PAD outcomes in Model 1 and in all models with simultaneous inclusion of CIMT. On the other hand, CIMT was independently related to PAD outcomes only when analyzed as a continuous variable in Models 1 and 2, but not as a dichotomical variable, and it did not improve risk discrimination (except in Model 2). In the analyses where both variables were concomitantly included, only cf-PWV remained predictive of PAD development/progression, but their concomitant inclusion did improve risk discrimination in relation to the previous models. When included as dichotomical variables, only increased cf-PWV predicted PAD outcome. The best discriminative risk performance of all the Cox models examined was that with ambulatory BPs (Model 2) with further inclusions of continuous cf-PWV and CIMT (C-statistic: 0.76; 95% CI 0.71–0.82). In sensitivity analysis, excluding those 52 individuals with baseline ABI < 0.9 did not materially change any of the results. Also, excluding the participants with PAD outcomes in the first 2 years of follow-up did not change the results.

**Table 2:** Results of multivariate Cox regression analyses for the independent predictors of incidence/progression of peripheral artery disease

Independent covariates	Hazard ratio	95% CI	p-value
Baseline variables			
Model 1:			
Age (1 year increase)	1.03	1.01–1.06	0.017
Sex (male)	1.45	0.90–2.32	0.12
Diabetes duration (1 year increase)	1.03	1.00–1.06	0.037
Microvascular complications (present/absent)	1.78	1.00–3.15	0.047
HbA_1c_ (1% increase)	1.19	1.06–1.33	0.004
Office SBP (10 mmHg increase)	1.08	0.99–1.18	0.078
C-statistic = 0.707 (0.653–0.761)			
Model 2:			
Age (1 year increase)	1.04	1.01–1.07	0.005
Sex (male)	1.64	1.02–2.65	0.043
Diabetes duration (1 year increase)	1.04	1.01–1.07	0.016
Microvascular complications (present/absent)	1.71	0.97–3.03	0.064
HbA_1c_ (1% increase)	1.15	1.02–1.29	0.022
24-h SBP (10 mmHg increase)	1.30	1.13–1.49	< 0.001
C-statistic = 0.732 (0.682–0.782)*			
Cumulative mean values until 2nd ABI measurement or occurrence of a hard outcome			
Model 3:			
Age (1 year increase)	1.02	1.00–1.05	0.10
Sex (male)	1.68	1.03–2.75	0.038
Diabetes duration (1 year increase)	1.04	1.01–1.07	0.005
Macrovascular complications (present/absent)	1.72	0.98–3.01	0.057
Microvascular complications (present/absent)	2.00	1.12–3.57	0.019
Mean office SBP (10 mmHg increase)	1.31	1.12–1.53	0.001
Mean LDL-cholesterol (10 mg/dL increase)	1.15	1.02–1.29	0.018
C-statistic = 0.722 (0.667–0.777)			

Candidate variables to enter the baseline models were age, sex, BMI, diabetes duration, smoking status, physical activity, diabetic treatment (metformin and insulin), anti-hypertensive treatment (number and classes of drugs), baseline office (Model 1) and ambulatory (Model 2) systolic and diastolic BPs, macrovascular and microvascular complications at baseline, baseline HbA_1c_, serum lipids (HDL- and LDL-cholesterol and triglycerides), use of statins and aspirin, and C-reactive protein. In the cumulative mean values model (Model 3), baseline office BPs, HbA_1c_ and serum lipids were substituted for their respective cumulative mean values until 2nd ABI measurement or the occurrence of any hard outcome. Age, sex, and baseline ABI were included in all models regardless of their significance

*CI* confidence interval, *SBP* systolic blood pressure, *LDL* low-density lipoprotein

^*^Significant (p < 0.05) increase in C-statistic in relation to baseline Model 1

**Table 3:** Results of multivariate Cox regression for incidence/progression of peripheral artery disease including aortic stiffness (carotid-femoral pulse wave velocity) and common carotid intima-media thickness into the models

Covariates	Model 1^a^		Model 2^a^		Model 3^a^	
	HR (95% CI)	C-statistic (95% CI)	HR (95% CI)	C-statistic (95% CI)	HR (95% CI)	C-statistic (95% CI)
Separated inclusion						
cf-PWV (1 m/s increase)	1.11 (1.02–1.21)^‡^	0.725 (0.669–0.780)	1.10 (1.01–1.20)^‡^	0.745 (0.694–0.796)	1.10 (1.01–1.20)^‡^	0.738 (0.681–0.794)
cf-PWV > 10 m/s	1.73 (1.02–2.93)^‡^	0.709 (0.654–0.762)	1.62 (0.96–2.72)	0.734 (0.684–0.783)	1.56 (0.91–2.68)	0.721 (0.663–0.779)
CC-IMT (0.1 mm increase)	1.22 (1.04–1.44)^‡^	0.718 (0.653–0.782)	1.18 (1.00–1.38)^‡^	0.755* (0.699–0.812)	1.13 (0.95–1.34)	0.738 (0.675–0.800)
CC-IMT > 1.05 mm	1.28 (0.72–2.26)	0.706 (0.645–0.767)	1.07 (0.61–1.90)	0.750 (0.698–0.802)	1.03 (0.57–1.88)	0.728 (0.666–0.789)
Concomitant inclusion						
cf-PWV (1 m/s increase)	1.18 (1.06–1.32)^†^	0.732* (0.670–0.795)	1.17 (1.05–1.31)^†^	0.762* (0.707–0.817)	1.17 (1.05–1.30)^†^	0.751* (0.689–0.813)
CC-IMT (0.1 mm increase)	1.16 (0.98–1.37)		1.12 (0.94–1.32)		1.08 (0.90–1.29)	
cf-PWV > 10 m/s	2.06 (1.17–3.60)^‡^	0.715 (0.656–0.775)	1.89 (1.10–3.27)^‡^	0.755* (0.704–0.807)	1.96 (1.09–3.53)^‡^	0.736 (0.673–0.800)
CC-IMT > 1.05 mm	1.18 (0.66–2.14)		1.00 (0.55–1.81)		0.98 (0.53–1.83)	

Values are hazard ratios (95% confidence intervals) and C-statistics (95% confidence intervals)

*HR* hazard ratio, *CI* confidence interval, *cf-PWV* carotid-femoral pulse wave velocity, *CC-IMT* common carotid intima-media thickness

^†^p < 0.01; ^‡^p < 0.05

^*^C-statistic significantly (p < 0.05) greater than those in their respective models shown in Table 2

^a^Models 1, 2 and 3 are the same as in Table 2 with further inclusion of the aortic stiffness and carotid IMT parameters

## Discussion

This prospective long-term follow-up study performed in a middle-aged type 2 diabetes cohort investigated the associations of traditional and non-traditional cardiovascular risk markers, namely CIMT and aortic stiffness, with the development/progression of PAD. It has four main findings. First, it confirmed associations of some traditional risk factors, such as older age, male sex, higher BPs and LDL-cholesterol levels, with PAD outcomes. Second, we demonstrated also associations between longer diabetes duration, presence of microvascular complications (diabetic kidney disease and peripheral neuropathy) and baseline HbA_1c_ levels, with PAD development/progression. Third, CIMT and aortic stiffness were predictors of PAD outcomes, independent of these traditional risk factors. Finally, the specific inclusion into the predictive models of ambulatory BPs (instead of office BPs), and of these two preclinical markers of atherosclerosis (CIMT and aortic stiffness), were capable of increasing PAD risk discrimination in relation to their precedent models, as assessed by the significant increases in C-statistics of the models.

Age, smoking, systolic blood pressure and cholesterol are established risk factors for PAD in general populations [1]. In diabetes these associations were less demonstrated [7, 10]. Dissimilar from ours and other studies [7, 10], some did not find relationships between LDL-cholesterol [8, 9] and arterial hypertension [9] and PAD development. Also, in contrast to our findings, the BARI 2D and ADVANCE studies did not find associations between lipid parameters and PAD development [13, 14]. In the BARI 2D trial [13], LDL- and HDL-cholesterol levels were predictive of PAD only as time-varying covariates (similar to our mean cumulative levels) in the subgroup of patients treated with insulin-sensitizing drugs. Like the present study, older age and longer diabetes duration were factors associated with PAD incidence in the UKPDS [7] and in a large study with Asian individuals from a tertiary diabetes care center [12]. Similar to some investigations [7, 12, 13], but different from others [10, 14], we observe associations between glucose control (baseline HbA_1c_ levels) and PAD development. The reasons for these contrasting results are not clear, but they may involve differences in studied populations, in the definitions of PAD outcomes or in data analyses with different multivariate adjustments.

Chronic kidney disease (CKD) measures, either increased albuminuria or decreased eGFR, have been shown to confer increased risks of incident PAD in a recent individual-participant meta-analysis [30]. These associations were also observed in individuals without diabetes or hypertension, suggesting that both measures might contribute to PAD incidence and not only represent end-organ damage from these diseases [30]. Oxidative stress, inflammation, activation of the renin-angiotensin system, endothelial dysfunction, abnormal calcium-phosphate metabolism, and elevation of lipoprotein(a) are among the feasible mechanisms connecting CKD and PAD incidence [31, 32]. However, independent associations between microvascular complications and PAD development have been scarcely demonstrated in diabetes [14]. Baseline macroalbuminuria and diabetic retinopathy (defined by previous retinal photocoagulation) were associated with PAD incidence in individuals from the ADVANCE study [14]. In the present study, the presence of diabetic kidney disease at baseline, defined either by increased albuminuria or by reduced eGFR, was associated with PAD development/progression. On the other hand, we did not observe any relation between the presence of retinopathy at baseline, nor with its severity, and PAD incidence/progression, in contrast to a recent report [33]. Possibly different definitions of PAD outcomes might explain this disparity. Otherwise, we found associations of PAD incidence/progression with the presence of peripheral neuropathy at baseline. Indeed, peripheral neuropathy has been associated with a greater risk of lower limb amputation in diabetes [34]. On the other hand, we had previously demonstrated that a baseline low ABI in asymptomatic individuals was predictive of future development/progression of peripheral neuropathy [4]. Hence, the pathophysiological mechanisms underlying the associations between PAD and peripheral neuropathy in type 2 diabetes might be bidirectional, possibly involving shared pathways such as insulin resistance, oxidative stress and endothelial dysfunction [35, 36].

CIMT, which is considered a marker of subclinical atherosclerosis, has been previously associated with PAD incidence in the Multi-Ethnic Study of Atherosclerosis (MESA) [37]. However, although CIMT has been demonstrated as a predictor of cardiovascular events in diabetic individuals [18, 38], the improvement in risk prediction after addition of CIMT to conventional risk factors is small [18], similar to that in the general populations [37, 39]. Nonetheless, only the Atherosclerosis Risk in Communities (ARIC) study investigated CIMT as a risk factor of PAD incidence specifically in a population sample of individuals with diabetes [5]. They demonstrated, like us, that CIMT was associated with PAD incidence after multivariate adjustment.

Otherwise, the most novel finding of this study was the demonstration of independent associations between increased aortic stiffness and PAD incidence/progression. Up to now, no study investigated prospectively associations between PAD incidence and increased aortic stiffness, only small cross-sectional studies explored these associations [40, 41], and none was performed in diabetic individuals. Aortic stiffness has been suggested as an compound marker not only of the effects of ageing and genetic background, but also of the additive jeopardizing results of cardiovascular risk factors on the arterial wall overtime [42]. Indeed, increased aortic stiffness has been previously demonstrated as a cardiovascular risk marker independent of conventional risk factors in meta-analyses of several clinical settings [43], as well as specifically in diabetic individuals [16, 17]. Here, we demonstrated that increased aortic stiffness, as assessed by its gold standard cf-PWV measurement [15], not only predicted future PAD development/progression, but also improved risk discrimination over a standard risk factor model.

This study has some limitations to notice. First, although it included a relatively large sample of individuals with a long follow-up, we did not observe a great number of PAD outcomes, what precludes a more comprehensive analysis. Second, it is a prospective observational cohort; hence no causal relationships, nor physiopathological inferences, can be made, but only speculated. Furthermore, some residual confounding might still have remained. Third, we did not evaluate annual ABI changes as a PAD outcome, but only a second ABI measurement performed after a median of 7.5 years after the baseline measurement. Hence, we could not establish the precise date of ABI reduction. Even so, we used the time-interval between ABI examinations as the time-to-event in the Cox analyses (except in participants with hard PAD outcomes), which adjust for the possible bias that a longer time-interval between ABI examinations might have increased the chance of having a larger ABI reduction. Forth, by protocol, we excluded from cohort entry patients with advanced renal failure (eGFR < 30 mL/min/1.73 m^2^), hence we cannot evaluate the impact of advanced CKD on PAD outcomes. Otherwise, we demonstrate that moderate diabetic CKD, defined by either having abnormal albuminuria (≥ 30 mg/24 h) or decreased eGFR (between 60 and 30 mL/min), was an important predictor of PAD outcomes. Finally, this study included middle-aged to elderly type 2 diabetic individuals followed at a tertiary-care center, so results may not be generalizable to other diabetic populations treated at primary care. However, this study has also some strengths to regard. It is a well-documented large cohort of patients with type 2 diabetes with annual outcomes evaluation over a long-term standardized follow-up, and a comprehensive investigation of the associated risk markers of PAD incidence/progression was performed, including CIMT and aortic stiffness.

## Conclusions

We demonstrated in a large type 2 diabetes cohort with a long-term follow-up that some traditional cardiovascular risk factors (older age, smoking, high BP and LDL-cholesterol), and some diabetes-related factors (longer diabetes duration, presence of macro- and microvascular complications, particularly CKD and peripheral neuropathy) were associated with PAD incidence/progression. Furthermore, 2 markers of subclinical atherosclerosis, CIMT and aortic stiffness, added prognostic information over and beyond these risk factors of PAD development, and they shall be considered/added in PAD risk stratification of individuals with type 2 diabetes.

## Data Availability

The Rio de Janeiro type 2 diabetes cohort study is an on-going study, and its dataset is not publicly available due to individual privacy of the participants. However, it may be available from the corresponding author on reasonable request.

## Abbreviations

ABI: Ankle-brachial index
PAD: Peripheral arterial disease
CIMT: Carotid intima-media thickness
SBP: Systolic blood pressure
cf-PWV: Carotid-femoral pulse wave velocity
eGFR: Estimated glomerular filtration rate
CKD: Chronic kidney disease
